# Differential changes in the effective neural drive following new motor skill acquisition between vastus lateralis and medialis

**DOI:** 10.1007/s00421-026-06239-0

**Published:** 2026-05-05

**Authors:** Caterina Cosentino, Hélio V. Cabral, Milena A. Dos Santos, Elmira Pourreza, J. Greig Inglis, Francesco Negro

**Affiliations:** 1 Department of Clinical and Experimental Sciences, Università degli Studi di Brescia, Viale Europa 11, 25121 Brescia, Italy; 2 School of Physical Education and Sports, Universidade Federal do Rio de Janeiro, Rio de Janeiro, Brazil; 3 Biomedical Engineering Program (COPPE), , Universidade Federal do Rio de Janeiro, Rio de Janeiro, Brazil; 4 Postgraduate Program in Rehabilitation Sciences, Universidade Federal do Rio de Janeiro, Rio de Janeiro, Brazil

**Keywords:** Short-term skill acquisition, Force control, HDsEMG, Discharge patterns, Synaptic input

## Abstract

**Purpose:**

To investigate whether short-term acquisition of a new motor task is mediated by changes in common synaptic inputs to motor neurons within and between synergistic muscles.

**Methods:**

Twenty one participants performed 15 repetitions of a complex force-matching task at 10% of maximal voluntary contraction. Two trials were selected for analysis, the one with the highest force-target error (pre-learning) and the one with the lowest (post-learning). High-density surface electromyograms recorded from vastus medialis (VM) and vastus lateralis (VL) were decomposed into motor unit spike trains, and units were tracked between trials. Motor unit discharge behavior and common synaptic oscillations across the delta, alpha, and beta bands were calculated and compared between pre- and post-learning.

**Results:**

Force-target matching improved across trials, accompanied by a significant decrease in discharge rate variability in VL (*p* < 0.001), while the mean discharge rate remained similar (*p* > 0.14). The area under the curve in delta band coherence decreased for VM (*p* = 0.01), VL (*p* < 0.001) and VM-VL motor units (*p* < 0.001). In contrast, alpha band decreased for VL (*p* < 0.001), but not for VM (*p* = 0.41). Notably, reductions in alpha band correlated significantly with performance improvements only in VL (*R* = 0.77) but not VM.

**Conclusion:**

The acquisition of a new motor task is mediated by modulations in common synaptic inputs to motor units, leading to improved force control. Our findings suggest muscle-specific associations between these changes in common synaptic inputs and short-term motor learning, particularly in the alpha band.

## Introduction

The acquisition of a new motor skill task is defined as the process by which movements become progressively more accurate through practice and interaction with the environment (Willingham 1998; Cheung et al. 2020; Doyon et al. 2003). This incremental process begins with a rapid initial phase, during which task repetition within a single session induces specific changes in movement control, ultimately enabling precise execution of the new motor skill (Doyon and Benali 2005). These early changes in movement control reflect neural plasticity at both cortical and subcortical levels, ensuring the coordinated activation of synergistic muscles required for the intended movement (Dayan and Cohen 2011; Ungerleider et al. 2002; Ziemann et al. 2004; Stefan et al. 2006; Aizenstein et al. 2004; Hikosaka et al. 2002; Doyon et al. 2002; Floyer-Lea and Matthews 2004; Cheung et al. 2020). For instance, the early learning phase is dominated by cerebellar activity, with peak activation of the primary motor cortex linked with error correction (Lohse et al. 2014). Thus, research on functional and neural circuitry adaptations at the supraspinal level has significantly advanced our understanding of motor skill learning mechanisms.

Spinal mechanics underlying voluntary movement generation have also been explored in the context of motor skill learning (Ely et al. 2022; Kinany et al. 2023; Vahdat et al. 2015; Landelle et al. 2021). Neural inputs transmitted from spinal and supraspinal sources to spinal motor neurons consist of independent synaptic inputs, as well as synaptic inputs shared across the entire pool (Negro and Farina 2012; Castronovo et al. 2018; Enoka and Farina 2021; Farina and Negro 2015). Due to the spread of shared inputs, the motor neuron pool functions as a selective linear filter, canceling out in the neural drive input components that are not common to all motor neurons. As a result, the cumulative spike train of action potentials received by the muscle (Farina and Negro 2015), is primarily determined by the common synaptic inputs (Hug et al. 2023 a; Negro et al. 2016; Thompson et al. 2018; Farina et al. 2014; Negro et al. 2009). Therefore, investigating changes in the neural drive provides direct information on alterations in the strength of common synaptic inputs to alpha motor neurons. Building on this concept, a recent study investigated how the acquisition of a force-matching task influences the low-frequency oscillations of shared synaptic inputs to alpha motor neurons (Cabral et al. 2024), which are key determinants of force generation and variability (Enoka and Farina 2021; Hug et al. 2023 a; Farina and Negro 2015; Negro et al. 2009). The findings revealed that the short-term acquisition of the task was associated with reductions in physiological tremor oscillations within the shared synaptic inputs, with these reductions correlating with improvements in performance. Additionally, compelling evidence suggested that learning a new motor task may modulate presynaptic inhibition by reducing H-reflex excitability, potentially facilitating motor sequence consolidation within spinal circuits (Lungu et al. 2010; Giboin et al. 2020; Perez et al. 2005). However, previous research has primarily focused on individual muscles controlling a single joint. How shared synaptic inputs are transmitted across synergistic muscles and modulated during skill acquisition remains unclear.

Therefore, the aim of this study was to investigate whether the short-term acquisition of a motor task is mediated by changes in the common synaptic inputs to motor neurons of synergistic muscle. We investigated this issue in the vastus lateralis (VL) and vastus medialis (VM) muscles, which act synergistically with the rest of the quadriceps group during knee extension. This synergistic pair is particularly relevant because VM and VL are believed to receive a high proportion of shared synaptic input during voluntary isometric contractions (Laine et al. 2015). However, they are anatomically and functionally distinct. Due to differences in muscle architecture (Flandry and Hommel 2011; Blazevich et al. 2006), the VL has a greater potential for force generation (Lin et al. 2004; Lieber et al. 2000). Therefore, we consider it relevant to investigate whether these architectural and functional differences would lead to distinct modulation of common synaptic inputs during the short-term acquisition of a motor task. We hypothesized that the acquisition of a new skill training task would be similar across synergistic muscles and that the effects of neural drive oscillations on the overall force output would mainly depend on the different anatomical conformation of the muscles, the mechanical constraints, and the functions they perform.

## Methods

### Participants

Twenty one healthy individuals (15 males and 6 female, age: 29 ± 6 years; height: 178 ± 11 cm; weight: 73 ± 18 kg) were included in this study. All participants were right-leg dominant and were free from neuromuscular pathologies or conditions affecting the lower limb. This study was conducted following the approval of the local ethical committee (number NP5665) and in accordance with the latest Declaration of Helsinki. Before the beginning of experiments, all participants provided written informed consent.

### Experimental protocol

**Fig. 1:**
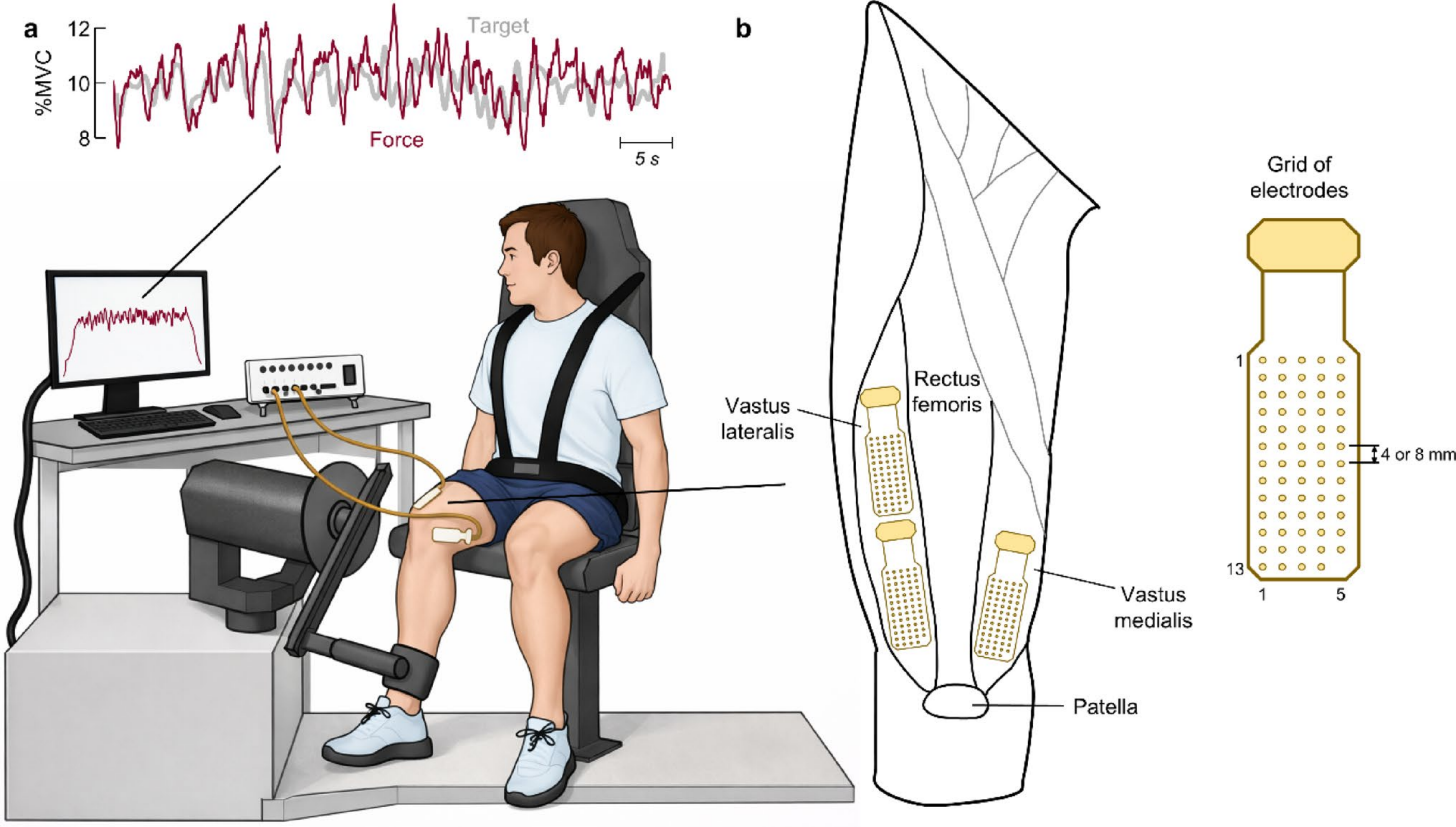
Experimental Setup **a** Participant’s position in the isokinetic dynamometer for the recording of the isometric knee extension task. Participants were seated on the dynamometer chair with the hip and knee joints positioned at 90° of flexion (0° being fully extended). The learning task consisted of following a randomly generated target signal, which oscillated above and below 10% of maximal voluntary contraction (gray thick line on the top panel). Visual feedback of the force (dark red line on the top panel) was displayed together with the target. **b** Schematic representation of the placement of electrode grids over the vastus lateralis and vastus medialis muscles. Three grids of 64 electrodes arranged in 13 rows x 5 columns were used

A single experimental session was performed, lasting approximately 90 min. Participants were seated in an isokinetic dynamometer (HUMACNORM Extremity System, CSMi Solutions, Stoughton, MA) with hip and knee joints positioned at 90° of flexion (0° being fully extended) (Fig. 1a). The ankle was secured three centimeters above the malleolus to assess isometric knee extension force.

First, participants performed three five-second maximal voluntary contractions (MVCs). To ensure that contractions were not influenced by fatigue, a three-minute rest period was provided between each MVC (Inglis and Gabriel 2021). The maximal force across the three MVCs was used to calculate the force level for the subsequent submaximal contractions (%MVC). Following three minutes of rest, participants performed 15 trials of a complex force-matching task at 10% MVC. The complex force-matching task consisted of a ramp-up phase at a rate of 10% MVC/s, a 30 s phase of a stochastic force oscillation, and a ramp-down phase at a rate of 10% MVC/s. During the 30 s stochastic force phase, subjects tracked a randomly generated signal with a frequency content below 1.5 Hz (−3 dB low pass frequency), which oscillated above and below 10% MVC (gray thick line on the top of Fig. 1a). The same trajectory was used throughout all trials and for all the participants. This protocol was based on prior studies (Knight and Kamen 2004; Cabral et al. 2024), which demonstrated clear performance improvement in a similar protocol, quantified through a reduction in the root-mean-square error (RMSE) between the force and target signals, across the 15 repetitions. Visual feedback of the force output was displayed on a monitor along with the target force trace (Fig. 1a). At the end of the protocol, two additional MVCs were performed to verify that there was no decline in maximal force production, as an indication of fatigue. No differences were found between the MVCs recorded at the beginning and the end of the protocol (before: 354.25 ± 160.85 kg; after: 380.01 ± 230.24 kg; Wilcoxon signed-rank test; *P* = 0.24).

### Data collection

During all 15 trials, the myoelectric signals from vastus medialis (VM) and vastus lateralis (VL) were recorded using high-density surface electromyography (HDsEMG). Specifically, three grids of 64 electrodes arranged in 13 rows x 5 columns (1 mm diameter, 8 mm inter-electrode distance, GR08MM1305, OT Bioelettronica, Turin, Italy) were placed over the distal VM region and the distal and proximal VL regions (Fig. 1b). The position of the grids was determined following Barbero et al. (2012). Following a preliminary analysis that revealed a very low number of extracted motor units from the VM muscle (see Results section), for the last 6 participants, three grids of 64 electrodes with smaller inter-electrode distance (4 mm) were applied over the VM, as recent evidence suggests that a larger number of close-spaced electrodes may provide a greater motor units yield (Caillet et al. 2023). Reference electrodes (WS1.1, OT Bioelettronica, Turin, Italy) were positioned on the right patella and the right ankle. Prior to electrode placement, the skin was shaved and mildly abraded (EVERI, Spes Medica, Genova, Italy) and cleaned with water to minimize skin impedance. Double-sided adhesive foam (FOA08MM1305 and FOA04MM1305, OT Bioelettronica, Turin, Italy) was used to attach each matrix to the skin. A thin layer of conductive paste (AC cream, OT Bioelettronica, Turin, Italy) was spread over the electrodes to ensure electrode-skin contact. The myoelectric signals were acquired in a monopolar configuration with a sampling frequency of 2048 Hz using a 16-bit amplifier (10–500 Hz bandwidth; Quattrocento, OT Bioelettronica, Turin, Italy).

### Data analysis

The analyses were conducted using customized MATLAB scripts (R2023b).

**Fig. 2:**
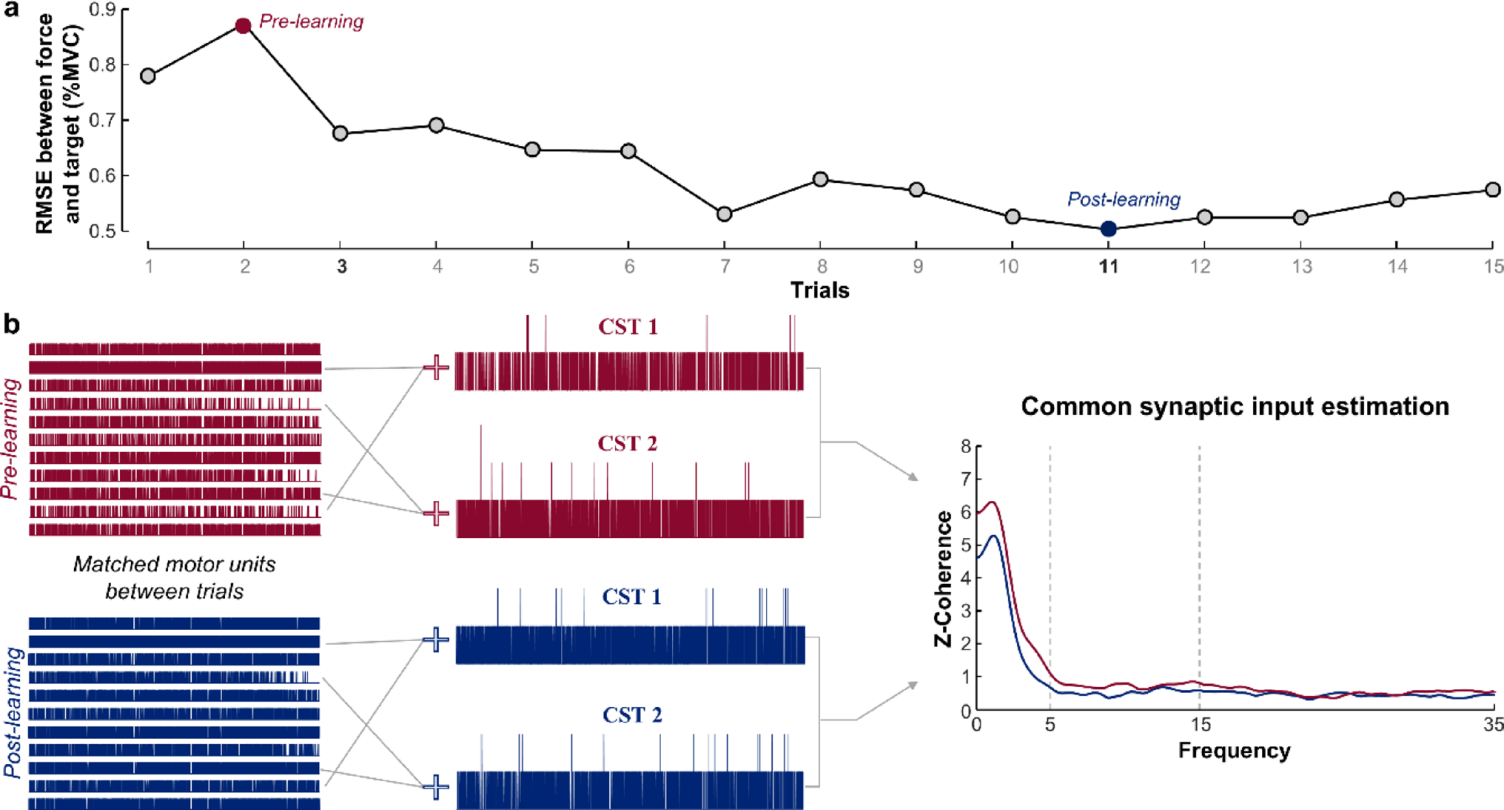
Common synaptic input estimation **a** Representative case of the root-mean-square error (RMSE) between force and target across the 15 trials of the learning task. Two trials were selected for analysis: the trial with the highest RMSE value (pre-learning; red circle) and the trial with the lowest RMSE value (post-learning; blue circle). **b** Motor units were decomposed and tracked between the pre-learning (red) and post-learning (blue) trials. Common synaptic oscillations were estimated through coherence analysis between two equally sized cumulative spike trains obtained by summing the discharge times of randomly selected pairs of motor units

*Force*: The force signals were initially low-pass filtered using a third-order Butterworth filter with a cutoff frequency of 15 Hz. Subsequently, during the 30 s force oscillation phase, the RMSE, and the cross-correlation between the target profile and the force were computed for all 15 trials. To evaluate improvements in task performance, as a measure of the short-term force-matching skill acquisition, the RMSE between the produced force and the target was calculated. Then, two out of the 15 trials were selected for analysis based on the RMSE values of each participant: the one with the highest (pre-learning) and lowest (post-learning) RMSE values between force output and target traces (Fig. 2a). The trials selection was based on a recent paper from our group (Cabral et al. 2024) showing that, when using the same protocol, there were no statistical differences in force-target RMSE values within both the initial two and the last two trials. Then, to maximize the number of matched motor units that could be reliably tracked before and after skill acquisition, we kept only one initial trial and one final trial. Specifically, the trials selected represented the highest (pre-learning) and lowest (post-learning) RMSE between force output and the target trace (Fig. 2a). To assess changes in force steadiness with the force-matching skill acquisition, the coefficient of variation of the force was calculated as the ratio of the standard deviation to the mean value during the 30-s force oscillation phase (Laidlaw et al. 2000). Finally, to examine whether participants temporally shifted their force output to match the target skill better, the time lag at which the cross-correlation with the target peaked was compared between pre- and post-learning trials.

#### Motor unit decomposition

HDsEMG signals were initially bandpass filtered using a third order Butterworth filter with cut-off frequencies between 20 and 500 Hz. Subsequently, signals were visually inspected by an expert operator, and noisy channels resulting from electrode contact issues or artifacts were excluded. The signals during the middle 30 s of the target (oscillatory region) were then fully decomposed using a convolutive blind source separation algorithm (Negro et al. 2016). This method allows for the automatic extraction of motor unit spike trains from the HDsEMG signals. The decomposition was performed independently on each electrode grid. Only motor units with a silhouette (SIL) of at least 0.87 were considered for further analyses. The SIL is a measure of accuracy of the decomposition that assesses the separability between identified motor unit spikes and noise (Negro et al. 2016). The same expert operator visually inspected all the identified motor units. Any missed or misidentified pulses (inter-spike intervals < 20 ms or > 250 ms (Negro et al. 2009) were manually added or removed based on the changes in the innervation pulse trains and SIL values of motor units. After the manual optimization of the extracted motor units, the occurrence of the same motor unit being recorded by different grids placed on the same muscle (e.g., proximal and distal VL regions) was assessed. In cases, duplicated units were identified across grids (i.e., motor units that shared more than 30% of their discharge times), the motor unit with the highest coefficient of variation of the inter-spike interval value was excluded for the subsequent analyses.

#### Motor unit tracking

Following the decomposition, the identified motor units were tracked between the pre- and post-learning trials by reapplying the motor unit separation vectors from the pre-learning to the post-learning trial and vice-versa (Oliveira and Negro 2021; Cabral et al. 2024). This procedure was performed separately for each electrode grid. For subjects who did not have at least four units tracked between trials (*n* = 8), the signals were further inspected to track the motor units based on the similarity of their shape (i.e., motor unit template matching method) (Martinez-Valdes et al. 2017). For this analysis, only motor units with a cross-correlation between their action potentials greater than 0.8 were considered (Martinez-Valdes et al. 2017). For all tracked motor units between trials, the mean discharge rate and the coefficient of variation of the inter-spike interval were calculated.

#### Common synaptic input within and between muscles

To assess changes in common synaptic inputs to the motor neuron pool following the short-term acquisition of a new motor skill task, coherence analysis was performed between motor unit spike trains (Castronovo et al. 2015; Negro and Farina 2012). Because of the summation process when converting synaptic inputs to the neural drive to the muscle, the motor neuron pool tends to cancel the noncorrelated components of the synaptic inputs so that only correlated components (i.e., common synaptic inputs) remain in the ensemble of the output spike trains (Farina et al. 2014; Dideriksen et al. 2012; Negro et al. 2009). In other words, the motor neuron pool acts as an effective linear filter (Farina and Negro 2015) and, thus, linear methods such as coherence analysis can be used to estimate the strength of the common synaptic inputs (Negro and Farina 2012). This analysis measures the linear coupling between two signals in the frequency domain, where zero indicates no coupling and one perfect coupling at a given frequency. Both within-muscle (VM and VL separately) and between-muscle (pooled VM-VL) coherence were performed. Changes in common synaptic oscillations were estimated separately for the delta (1–5 Hz), alpha (5–15 Hz), and beta (15–35 Hz) bands. The selection of these specific frequency bandwidths was driven by their physiological relevance in motor control. The delta band in motor unit coherence is thought to reflect oscillations associated with the voluntary control of force, as it contains the frequency range of the force output (Farina and Negro 2015). The oscillations within the alpha band are usually attributed to physiological tremor within the Ia afferent feedback loop (Christakos et al. 2006; Conway et al. 1995a) and can be viewed as common noise inputs that limit force accuracy. Finally, the beta band oscillations are believed to be of cortical origin, as significant corticomuscular coherence has been reported within this bandwidth (Baker et al. 1997; Conway et al. 1995b). Coherence analysis in these three frequency bands therefore provides information about common synaptic inputs to the alpha motoneuron pool arising from supraspinal and spinal pathways (Negro and Farina 2012). Specifically, for this study, the discharge times of motor units were converted into binary sequences (i.e., motor unit spike trains), and two equally sized cumulative spike trains (CSTs) were obtained by summing the binary sequences of two randomly selected motor units from the matched units (Fig. 2b) (Negro and Farina 2012; Rossato et al. 2022; Maillet et al. 2022; Castronovo et al. 2015). This process was repeated for up to 100 iterations and the average coherence was calculated for further analysis. For all iterations, coherence was calculated between the two detrended CSTs using Welch’s periodogram with a Hanning window of one second and an overlap of 95% (Fig. 2b). The averaged coherence values were converted into standard z-scores as suggested by Gallet and Julien (2011). Only z-coherence values greater than the bias, which was determined as the mean z-coherence between 250 and 500 Hz (Castronovo et al. 2015), were considered. To assess changes in the common synaptic inputs across different frequency bandwidths, the area under the curve was calculated for delta, alpha, and beta bands. Subsequently, the ratio of the area under the curve (post/pre) was calculated and subtracted by one. In this way, positive ratio values indicate an increase in coherence with the short-term skill acquisition, while negative values indicate a decrease.

#### Coupling between force/neural drive and target oscillations

To assess whether there were changes in the linear coupling between the oscillations in force and the target oscillations, as well as between oscillations in the neural drive (i.e., CSTs) and the target oscillations, coherence analysis between these signals was performed. This analysis was carried out similarly to the coherence between motor unit spike trains. However, as delta band oscillations contain the frequency range of the force output and, thus, are associated with voluntary force control, only the delta band was considered for this analysis. For both force-target and CST-target coupling, an increase in the coupling indicates an enhancement in the representation of common synaptic input oscillations relevant to optimal force control in the intended motor task.

### Statistical analysis

Statistical analyses were performed in R (v. 4.3, R Core Team 2023) using the RStudio environment (v. 2023.03.1). Non-parametric tests were used considering the data was non-normally distributed (Kolmogorov-Smirnov test; *P* < 0.001 for all). The Wilcoxon signed-rank test was performed to compare the force-target RMSE, the force-target cross-correlation values and the force-target cross-correlation lags between trials. The Wilcoxon signed-rank test was also used to compare the coefficient of variation of force between the pre-learning and post-learning trials.

Linear mixed models (LMMs) were employed to compare the mean motor unit discharge rate and the coefficient of variation of the inter-spike interval between pre- and post-learning. LMMs were implemented with the *lmerTest* package (Kuznetsova et al. 2017) using the Kenward-Roger method to approximate the degrees of freedom and estimate *P*-values. For this analysis, random-intercept models were used, considering time (pre-learning and post-learning), as a fixed effects and participant as a random effect. The marginal means with 95% confidence level intervals were estimated using the *emmeans* package (Lenth 2016). To compare z-coherence values between pre- and post-learning trials, changes in the area under the curve ratio (post/pre) were assessed using the one-sample Wilcoxon test (null hypothesis µ_0_ = 0), separately for each frequency band. To determine whether the changes in the area under the curve of the z-coherence were associated with the number of motor units, Pearson’s correlations were performed between the number of matched motor units and the area under the curve ratios, separately for the delta and alpha bands. For this analysis, the VM and VL results were pooled together. To evaluate changes in the area under the curve ratio of the force-target z-coherence and CST-target z-coherence, the one-sample Wilcoxon test was used.

Separately for each muscle, repeated measures correlations were performed between the force-target RMSE and the area under the curve ratio within the delta and alpha bands to test whether the observed reductions in delta and alpha band coherence (see *Results*) were correlated with performance improvements. These analyses were performed using *rmcorr* package with fixed slopes (Bakdash and Marusich 2017). Considering the limited number of participants, bootstrapping was used in order to report the 95% confidence interval and obtain bias-corrected intervals. A total of 1,000 iterations were performed, each using 75% of the sample size (10 participants). To compare the effect of muscle on the relationship between z-coherence–RMSE in the delta and alpha frequency bands, a LMM was performed with RMSE as the dependent variable and subject as a random effect. The area under the curve (delta or alpha band), muscle (VM or VL), and their interaction were included as fixed effects. For all the variables, statistical significance was set at an alpha of 0.05. All individual data of motor unit discharge times for both the VM and VL acquired in the pre- and post-learning trials are available at 10.6084/m9.figshare.28847492.

## Results

### Changes in force oscillations between pre- and post-learning

**Fig. 3:**
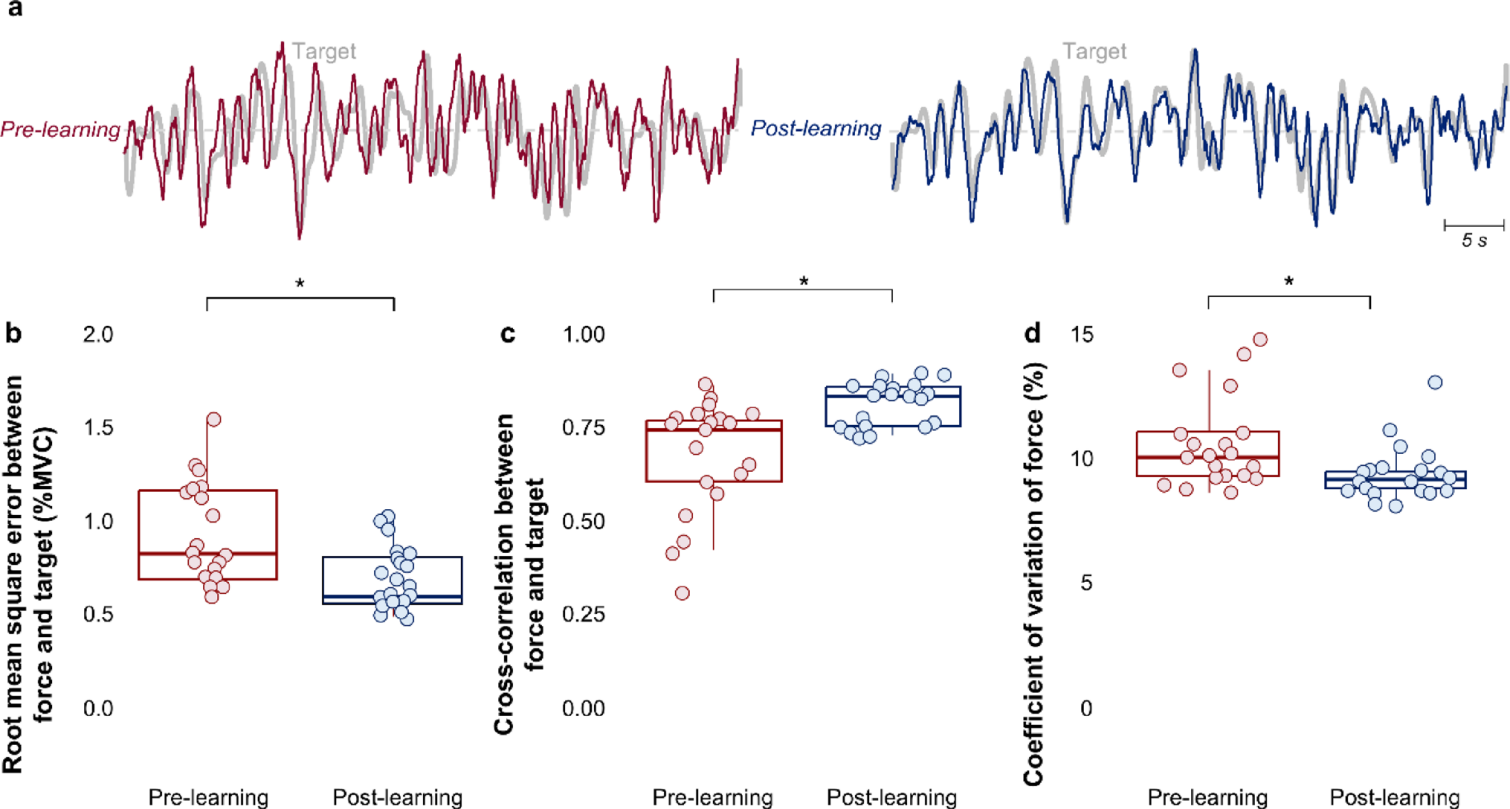
Force-target matching results **a** Representative case of the force during the pre-learning (red) and post-learning (blue) trials. The target is shown in gray. **b** Group results of the root-mean-square error between the force and the target. **c** Group results of the cross-correlation between the force and the target. **d** Group results of the coefficient of variation of force. Circles identify individual participants. Horizontal traces, boxes, and whiskers denote the median value, interquartile interval, and distribution range. *p < 0.05

To assess improvements in force-matching between pre- and post-learning trials, the RMSE and cross-correlation values between the force output and the target trace were calculated. Figure 3a shows the force traces of a representative participant during the pre-learning (red) and post-learning (blue) trials. It can be visually observed that there is a greater overlap between the force and the target (gray line) in the post-learning compared to the pre-learning, indicating improvements in performance.

These observations were confirmed by the group results. There were statistically significant decreases in RMSE (Fig. 3b; *p* < 0.001) and increases in the cross-correlation (Fig. 3c; *p* < 0.0001) between the force and the target when comparing post-learning with pre-learning trials. However, the cross-correlation lags did not significantly change between the pre- and post-acquisition trials (*p* = 0.65). Moreover, to assess changes in force steadiness, the coefficient of variation of the force was calculated and compared between trials. As shown in Fig. 3d, there was a significant decrease in the coefficient of variation of force (*p* < 0.001), which indicates an increase in force steadiness with force-matching skill acquisition.

#### Motor unit discharge behavior

A total of 324 motor units were identified from the VM (164 pre-learning; 160 post-learning), with an average (± standard deviation) of 7 ± 4 motor units per participant in the pre-learning (6 ± 3, 3 ± 2 and 3 ± 2 in the three grids over the VM) and 7 ± 4 motor units in the post-learning (5 ± 3, 4 ± 2 and 4 ± 2 in the three grids over the VM). When comparing the number of motor units identified after changing the matrix configuration in the VM with the number identified initially, they were not significantly different (first matrix configuration: 6 ± 3 matched motor units per participant; second matrix configuration: 6 ± 3 matched motor units per participant; *P* = 0.83). In the VL, 927 motor units were identified (455 pre-learning; 472 post-learning), with an average of 21 ± 10 (14 ± 9 and 7 ± 3 in the two grids over the VL) and 21 ± 11 (15 ± 9 and 8 ± 3 in the two grids over the VL) motor units per participant in the pre- and post-learning trials, respectively. These motor units were subsequently matched between pre- and post-learning trials, leading to an average of 6 ± 2 matched motor units for the VM (5 ± 2, 3 ± 1 and 3 ± 1 in the three grids over the VM) and 12 ± 8 for the VL (12 ± 8 and 5 ± 2 in the two grids over the VL). The average (± standard deviation) SIL values of the cleaned, matched motor units were 0.93 ± 0.04 for the VM and 0.95 ± 0.04 for the VL.

To assess changes in motor unit discharge behavior following the short-term acquisition of the force-matching skill task, the mean discharge rate and coefficient of variation of the inter-spike interval of tracked motor units were calculated. For both VM and VL, there were no significant changes in mean discharge rate between pre- and post-learning (VM: from 8.38 [7.86, 8.89] pps to 8.00 [7.46, 8.54] pps, *p* = 0.14; VL: from 8.27 [7.77, 8.77] pps to 8.18 [7.67, 8.68] pps, *p* = 0.44). In contrast, the coefficient of variation of the inter-spike interval significantly decreased between trials for the VL only (from 40.9 [36.3, 45.5] % to 26.4 [21.8, 31.0] %, *p* < 0.0001), but not for the VM (from 38.9 [32.2, 45.7] % to 32.3 [25.3, 39.4] %, *p* = 0.08).

#### Common synaptic input oscillations

**Fig. 4:**
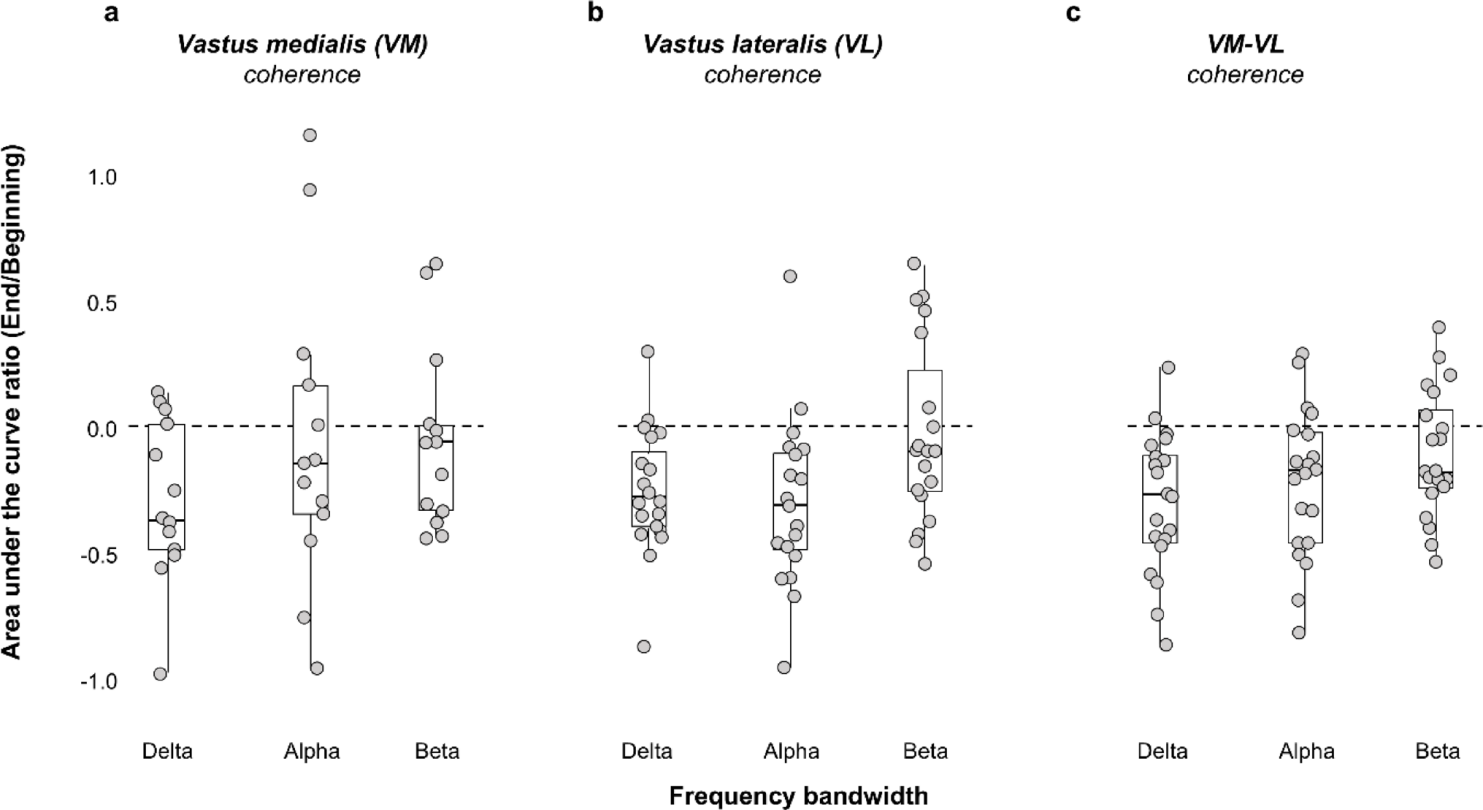
Motor unit coherence results Group results of the area under the curve ratio of coherence for VM (**a**), VL (**b**), and pooled VM-VL muscles (**c**). Three frequency bandwidths were analyzed: delta (1–5 Hz), alpha (5–15 Hz), and beta (15–35 Hz). Median value, interquartile range, and distribution range are denoted by the horizontal traces, boxes, and whiskers, respectively. Circles represent individual participants

To evaluate alterations in common synaptic inputs, the area under the curve ratio (post/pre) was calculated for the delta (1–5 Hz), alpha (5–15 Hz), and beta (15–35 Hz) bands. While delta band oscillations in motor unit coherence are linked with changes in voluntary force control, changes in alpha band are commonly associated with physiological tremor oscillations that hampers force control (Christakos et al. 2006). Finally, alterations in beta band are connected to changes in corticomuscular coupling (Baker et al. 1997). Only participants with at least four tracked motor units between trials were included in the study (see Methods); thus, 13, 19 and 20 participants were included for VM, VL and VM-VL coherence, respectively. In both muscles, significant reductions were observed in the area under the curve within the delta band (*p* = 0.005 for VM; *p* < 0.001 for VL). In the alpha band, a significant decrease was observed only in VL (*p* < 0.001), but not VM (*p* = 0.41). In the beta band, neither muscle showed significant differences between pre- and post-learning (*p* = 0.79 for VM; *p* = 0.57 for VL) (Fig. 4a and b). Similar to the results in VL, significant VM-VL coherence decreases were observed in the delta and alpha bands with the force-matching skill acquisition (*p* < 0.01 for both), but not for the beta band (*p* = 0.06; Fig. 4c). To assess if observed changes in common synaptic oscillations were associated with the number of included motor units, we performed a Pearson’s correlation analysis between the area under the curve ratio (post/pre) and the number of matched motor units. For both the delta (*R* = −0.06, *p* = 0.76) and the alpha bands (*R* = −0.17, *p* = 0.36), the number of motor units was not significantly correlated with changes in the area under the curve.

**Fig. 5:**
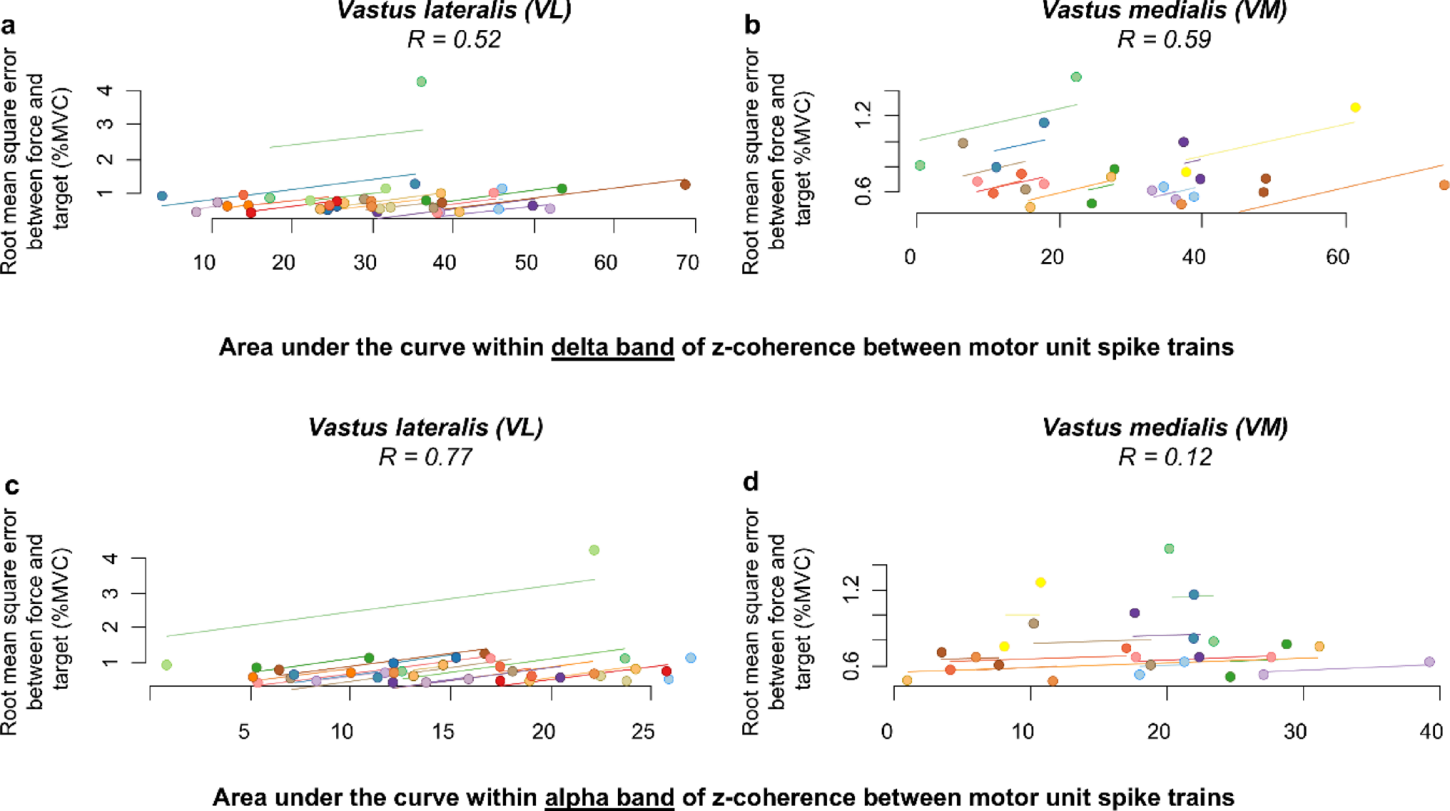
Correlation results Repeated measures correlation between the force-target root-mean-square error and the area under the curve of motor unit z-coherence within the delta band (**a** and **b**), and the alpha band (**c** and **d**). The left panel shows the results for the vastus lateralis muscle and the right panel for the vastus medialis muscle

To investigate whether reductions in motor unit z-coherence within the delta and alpha bands were associated with improvements in the force-matching task, we applied a repeated measures correlation. For both bands, a significant positive correlation was observed between the force-target RMSE and the z-coherence in the VL (delta: *R* = 0.52 [0.47, 0.66]; Fig. 5a; alpha: *R* = 0.77 [0.66, 0.82]; Fig. 5c). In contrast, for the VM, only the correlation between the force-target RMSE and the z-coherence in the delta band was significant (delta: *R* = 0.59 [0.21, 0.85]; Fig. 5b; alpha: *R* = 0.12 [−0.23, 0.39]; Fig. 5d). LMM analysis revealed a significant interaction between muscle and alpha-band z-coherence (*p* < 0.01), indicating stronger within-subject associations between z-coherence and force-target RMSE in VL compared with VM. In contrast, there was no significant muscle and z-coherence interaction for delta-band (*p* = 0.37).

#### Linear coupling between force/neural drive and target oscillations

**Fig. 6:**
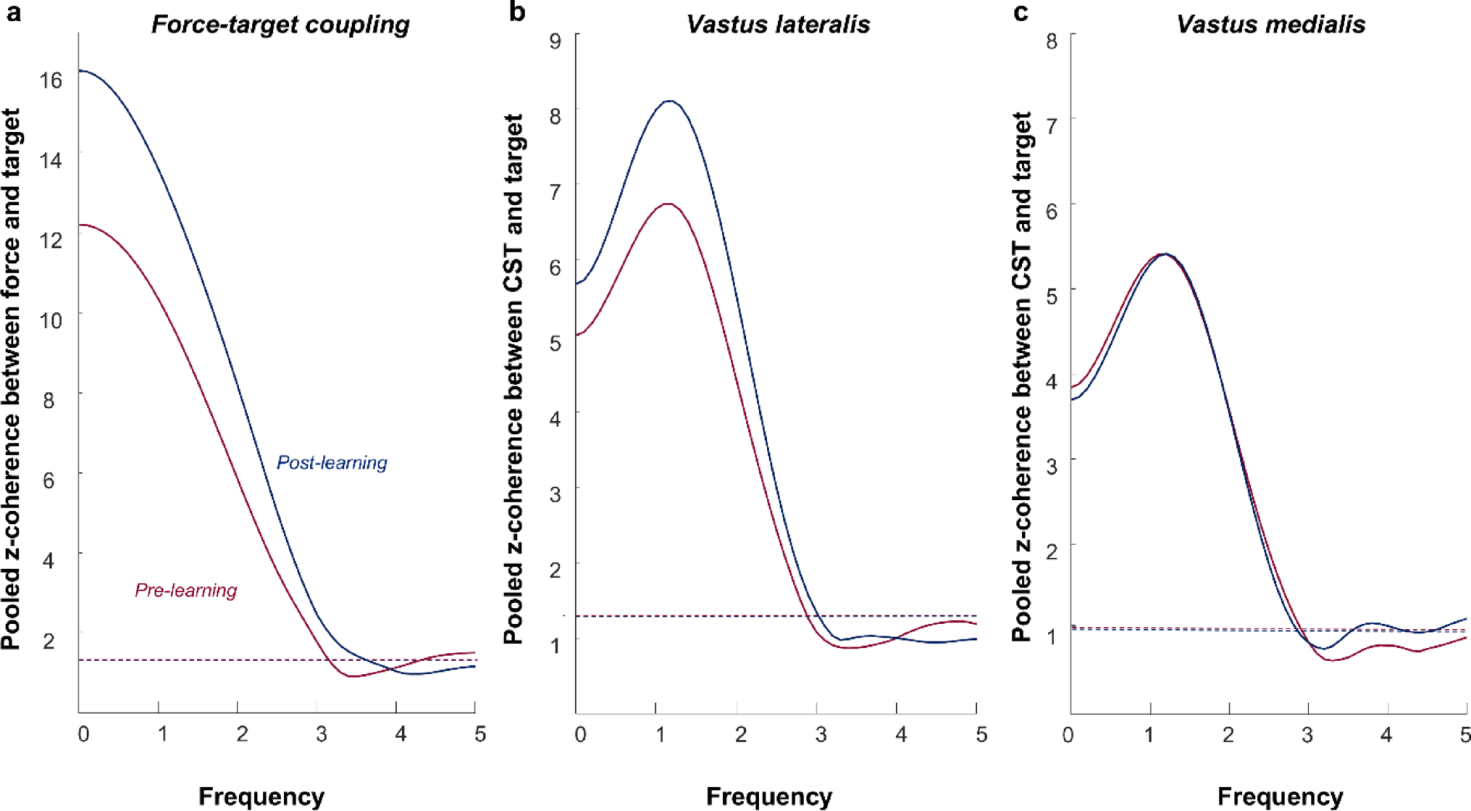
Coherence results between force/neural drive and target oscillations The pooled z-coherence profiles between force and target (**a**) and cumulative spike train (CST) and target (**b** and **c**) are shown. The CST-target coherence analysis was performed separately for the vastus lateralis (**b**) and vastus medialis (**c**) muscles. The red and blue lines denote the pre-learning and post-learning trials, respectively

Changes in the linear coupling between the force/neural drive oscillations and the target trace oscillations were investigated using coherence analysis between these signals within the delta band. Figure 6 shows the pooled z-coherence (i.e., average across participants) between the force and target, and between the neural drive (i.e., CST) and the target for both VM and VL. For both force-target z-coherence (*p* < 0.001; Fig. 6a) and VL CST-target z-coherence (*p* = 0.03; Fig. 6b), there was a significant increase in the area under the curve between pre- and post-learning trials. In contrast, the z-coherence between the VM CST and target did not significantly change between trials (*p* = 0.53; Fig. 6c).

## Discussion

This study investigated changes in the common synaptic input to synergistic muscles (VM and VL) during the short-term acquisition of a novel isometric motor task. Our main findings demonstrated that force-matching performance improved with skill acquisition. These improvements were accompanied by a reduction in common synaptic oscillations to spinal motor neurons in the delta coherence band, both within and between synergistic muscles, and in the alpha band for the VL and between synergistic muscles. Interestingly, reductions in alpha band oscillations were positively correlated with performance improvements, but only for the VL. As discussed below, these results suggest muscle dependent associations between changes in neural drive oscillations and short-term motor acquisition, underscoring the importance of considering the functional role of synergistic muscle.

The neural drive received by the muscle (i.e., the cumulative spike train of alpha motor neuron action potentials) is low-pass filtered by the contractile properties of active motor units at approximately 12 Hz (Windhorst 2012). Consequently, low-frequency oscillations in the neural drive (i.e., delta and alpha bands of motor unit coherence) play a determinant role in force control (Farina and Negro 2015; Hug et al. 2023 b). Therefore, we investigated whether short-term acquisition of a new force-matching task is mediated by changes in the effective neural drive to synergistic muscles, by assessing coherent oscillations in motor unit spike trains of VM and VL. Our results revealed that short-term skill acquisition was followed by reductions in alpha band coherence both in VL and between vastii motor units (Fig. 4). Considering alpha band (physiological tremor) oscillations are considered an involuntary noise component of the common synaptic inputs that negatively influence force accuracy (McAuley and Marsden 2000; Yavuz et al. 2015; Watanabe et al. 2018; Erimaki and Christakos 1999), their reduction during skill acquisition likely represents a neural mechanism to enhance force precision (Laine et al. 2014; Henderson et al. 2022; Williams et al. 2010; Koželj and Baker 2014). These findings support previous studies that demonstrated a strong link between reduced physiological tremor oscillations and improved force control (Novak and Newell 2017; Christakos et al. 2006; Williams et al. 2010; Koželj and Baker 2014; Laine et al. 2016). Although the precise mechanisms underlying alpha band reduction during short-term acquisition remain unclear, spinal interneurons phase-cancelling descending alpha oscillations have been proposed as a plausible mechanism (Cabral et al. 2024). Additionally, learning a new motor task has been shown to modulate presynaptic inhibition by reducing H-reflex excitability (Lungu et al. 2010; Giboin et al. 2020; Perez et al. 2005), which could also explain alpha band reductions. Finally, the reduction in alpha-band oscillations may be attributed to supraspinal mechanisms, particularly to attentional processes (Adrian and Matthews 1934; Pollen and Trachtenberg 1972; Hanslmayr et al. 2011; Klimesch 2012). Cortical alpha suppression has been considered a marker of enhanced processing of visual feedback (Bacigalupo and Luck 2022) and may be associated with observed alpha band reductions in motor unit coherence after short-term skill acquisition.

Interestingly, nonetheless similar results in coherence were observed between VL and pooled VM and VL motor units (see Fig. 4), only reductions in delta-band coherence were positively correlated with performance improvements in both muscles (Fig. 5a and b). In contrast, reductions in alpha-band coherence were positively correlated with performance improvements only in VL (Fig. 5c), but not VM (Fig. 5d). These differences in the correlations were confirmed by a linear mixed-effects model, which revealed a significant interaction between muscle and z-coherence on the alpha band–RMSE relationship, with the VL showing stronger associations than VM. Given that VM and VL are synergistic muscles that share most of their synaptic inputs (~ 75–95%; Laine et al. 2015), this finding was unexpected. However, architectural differences between these muscles may explain this discrepancy. Although both VM and VL are part of the same muscle group (i.e., quadriceps), their muscle architecture differs significantly (De Souza et al. 2018; Lieber and Fridén 2000; Ward et al. 2009; Lefebvre et al. 2006). The VL has a larger cross-sectional area, with muscle fibers arranged more transversely from the distal to the proximal region (Blazevich et al. 2006). These anatomical features provide the VL with a mechanical advantage for force production, while the VM primarily contributes to knee joint stabilization and force transmission to the tendon (De Souza et al. 2018; Lin et al. 2004; Lefebvre et al. 2006; Farahmand et al. 1998; Lieber et al. 2000). Thus, the positive correlation between alpha band reductions and performance improvements only in VL might suggest a mechanical advantage to produce force, which facilitates more efficient transduction of neural inputs into force. This interpretation was further supported by the CST-target coupling analysis, which revealed a significant increase in coupling between pre- and post-learning trials for the VL (Fig. 6b), but not for the VM (Fig. 6c). Therefore, while synergistic muscles such as the vastii share a significant portion of their synaptic inputs (Laine et al. 2015), their motor output is influenced by their distinct contractile properties and force-generating capacity. These findings highlight the importance of considering muscle-specific adaptations when investigating neural drive changes during motor short-term acquisition.

Our findings also revealed a decrease in common synaptic oscillations within the delta band following skill short-term acquisition, which plays a critical role in optimal force production and task execution accuracy (Negro et al. 2009). A plausible explanation for the delta band reductions observed in this study relates to the motor strategy adopted by participants during the short-term acquisition of the force-matching isometric knee extension task. In both pre- and post-learning trials, participants followed the target similarly in time, as confirmed by the absence of significant differences in force-target cross-correlation lags (see *Results*). Thus, improvements in force-matching accuracy were achieved by decreases in force amplitude oscillations, which was confirmed by significant decreases in the coefficient of variation of force. This reduction in force variability, together with the increased coupling between force and feedback across trials (Fig. 6a), explains the delta band coherence reductions.

A final consideration concerns alterations in motor unit discharge behavior during task short-term acquisition. Unlike previous studies (Knight and Kamen 2004; Patten and Kamen 2000; Cabral et al. 2024), no significant changes in mean discharge rate between pre- and post-learning trials were observed. This suggests that mean discharge rate alone may not explain the observed changes in common synaptic oscillations. Instead, the reduction in motor unit discharge variability between trials in VL, quantified by the coefficient of variation of the inter-spike interval, contributed to the observed changes in common synaptic oscillations. This aligns with previous findings demonstrating a correlation between reductions in common synaptic oscillations and decreases in the coefficient of variation of the inter-spike interval (Heckman et al. 2009). Consistent with these findings, numerous studies have suggested that improvements in force steadiness are primarily driven by modulation in the common drive to motor neurons, rather than changes in motor unit discharge rate (Semmler et al. 2006; Dideriksen et al. 2012; Bilodeau et al. 2000; Tracy et al. 2004; Marmon et al. 2011; Farina and Negro 2015).

There are a few limitations of this study that we would like to acknowledge. Although the results of this study suggest that short-term acquisition is associated with modulations in the shared synaptic inputs to alpha motor neurons, we acknowledge that due to the chosen protocol, these findings may not be generalized to overall motor learning. Existing literature highlights that retention and/or transfer tests must be conducted to refer to motor learning, as these allow for the evaluation of persistence beyond the within-session practice (Kim et al. 2021; Lage et al. 2024; Chung et al. 2021; Hung et al. 2021). Since such tests were not performed in this study, the results can only be interpreted as reflecting short-term acquisition. Moreover, the present study does not include a control condition to assess whether the observed changes in neural drive cannot be solely attributable to the repetition of the motor task independent of short-term acquisition. However, in a recent study of our group (Cabral et al. 2024), we showed that the repetition of fifteen steady isometric contractions (control condition) led to an increase in alpha band coherence, which is the opposite of what was observed during the short-term skill acquisition. These findings rule out the possibility that the observed changes in alpha band are only attributable to motor task repetition independent of acquisition. Furthermore, despite the absence of a correlation between the number of motor units and the coherence values, the significantly lower motor unit yield, particularly in VM, may have partly affected the robustness of the results. Thus, between-muscle differences should be interpreted with caution and future studies with larger samples should further investigate muscle-specific synergistic mechanisms underlying motor learning. Finally, although previous literature has highlighted that motor unit behavior differs between sexes (Lulic-Kuryllo and Inglis 2022), it was not possible to perform statistical comparisons between sexes in this study. This limitation is mainly due to the difficulty in decomposing and tracking motor units in females, which has already been reported in the literature (Taylor et al. 2022).

In conclusion, this study demonstrated that, similar to individual muscles, the acquisition of a new motor task is mediated by modulations in the common synaptic inputs to motor units, leading to improved force control and short-term motor acquisition. Notably, our findings suggest muscle-dependent associations between alpha band oscillations and improvement in force-target error, highlighting the importance of assessing muscle-specific adaptations in motor short-term acquisition.

## Abbreviations

CST: Cumulative spike train
HDsEMG: High-density surface electromyography
LMM: Linear mixed model
MVC: Maximal voluntary contraction
RMSE: Root-mean-square error
SIL: Silhouette
VL: Vastus lateralis
VM: Vastus medialis
